# Characterization of the complete mitochondrial genome of *Melibe japonica* (Eliot, 1913) collected from Korean water

**DOI:** 10.1080/23802359.2018.1501321

**Published:** 2018-10-25

**Authors:** Su-Jeong Yang, Hyunjung Seo, Seok-Gwan Choi, Sangdeok Chung, Hyun-Woo Kim

**Affiliations:** a Department of Marine Biology, Pukyong National University, Busan, Republic of Korea;; b National Institute of Fisheries Science (NIFS), Busan, Republic of Korea;; c Interdisciplinary program of Biomedical, Mechanical and Electrical Engineering, Pukyong National University, Busan, Republic of Korea

**Keywords:** Mitochondrial genome, *Melibe japonica*, Korea, mollusk, sea slug

## Abstract

*Melibe japonica* is the rarely identified nudibranch species in Korean and Japanese waters. Total mitochondrial genome of *M. japonica* collected from the coastal water of Busan, Korea, was determined by the bioinformatic assembly of the contigs generated by Illumina Miseq platform. The circular complete mitochondrial genome of *M. japonica* was 13, 216 bp in length, which contains 13 proteins, 2 ribosomal RNAs, and 22 tRNAs. Mitochondrial gene order of *M. japonica* is considerably different from the mitochondrial genome of *Melibe leonina* in which 6 genes (ND1, COX2, CYTB, ND4L, ND5, and ND6) were differently located. Phylogenetic analysis with COI regions showed that *M. japonica* is most closely related to *Melibe viridis*. However, not all *Melibe* species formed a single clade based on the phylogenetic analysis by COI region and more mitochondrial genomes in this genus should be further studied to have a better knowledge of the evolutional relationship of the nudibranchs.

*Melibe japonica* is the extremely rare nudibranch species, which has identified mostly in Korean and Japanese waters (Eliot, 1913). Although *M. japonica* is now considered a possible synonym of *Melibe viridis* according to World Register of Marine Species (WoRMS, http://www.marinespecies.org/), it’s larger body length (∼30 cm), the higher proportions of the oral hood, the reddish pigmentation, and the ceras over the dorsum makes it clearly distinguishable from *M. viridis.* We collected a live *M. japonica* from the coastal water in Taejongdae, Busan, South Korea (35.060642°N, 129.076001°E) on 28 September 2015 (https://www.youtube.com/watch?v=1_sMaDKr97Q). After morphological analysis, collected specimen has been stored at −70 C° freezer in Pukyong National University.

The genomic DNA was extracted using an E.Z.N.A^®^ Mollusc DNA Kit (Omega Bio-tek, Norcross, USA) following the manufacturer’s instructions. COI sequence of the collected *M. japonica* showed only 82% nucleotide sequence identity to *M. viridis* (GenBank number: HM162700). Total mitochondrial genome was determined by the bioinformatic assembly of two large PCR products (4.7 kb and 9.0 kb) using MiSeq platform (Illumina, San Diego, USA). Paired-end contigs were constructed using the Mothur software package v 1.35.0 (Schloss et al., 2009). The mitochondrial genome was constructed by assembling the contiguous sequences using Geneious R8 (Kearse et al., 2012). The mitochondrial genome sequence *M. leonina* (GenBank number: NC026987), the only published mitochondrial genome in genus Melibe, was used as a reference for the gene organization analysis (Sevigny et al., 2015). Phylogenetic tree was constructed by Molecular Evolutionary Genetics Analysis program (MEGA7; http://www.megasoftware.net) with the Minimum Evolution algorithm.

The circular complete mitochondrial genome of *M. japonica* (GenBank number: MF034146) was 13, 216 bp in length, which contains 13 proteins, 2 ribosomal RNAs, and 22 tRNAs. Conserved start codons (ATN) and stop codons (TAA, TAG) were identified in all the protein-coding genes except for ND2. Among the protein-coding genes, 1 and 10 nucleotides were overlapped between ND1-COX2 and ND5-ND6, respectively. The gene order of *M. japonica* is considerably different from *M.leonina* in which ND1, COX2, CYTB, ND4L, ND5, and ND6 genes are located at L strand and ATP6 and ATP8 genes are in the H strand. In addition, ND1 is located between 16S rRNA and COX2 and, ND5 and ND6 are located between ND4L and ATP6.

Due to the high variability in the gene order among molluscan species and the lack of the nudibranch complete mitogenome sequences (Boore and Brown, 1998; Burger et al., 2000), we constructed the phylogenetic tree based on the COI regions (Figure 1). Phylogenetic analysis showed that *M. japonica* was most closely related to *Melibe viridis*. Species in the order Nudibranchia were divided into two clades. Five *Melibe* species including *M. japonica* were clustered together but *M. leonine, Tritonia diomeda* and *Roboastra europaea* formed a different clade which showed more closely related to those in Pleurobrachomorpha suggesting the re-examination of their taxonomy (Figure 1). More mitochondrial genome sequences in this genus should be analyzed to know the evolutional relationship of nudibranchs.

**Figure 1.**
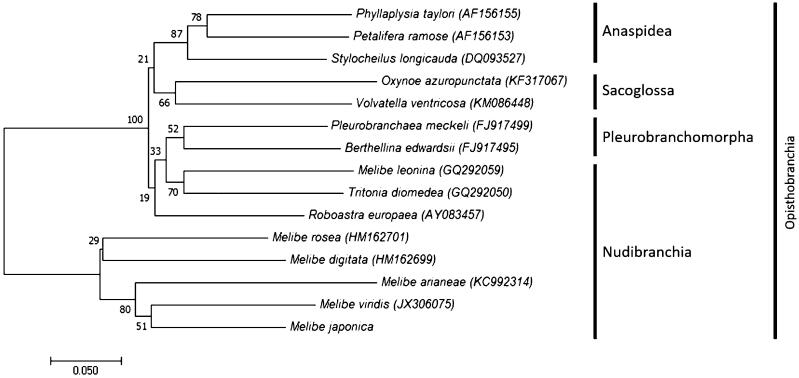
Phylogenetic tree of Melibe japonica constructed by COI sequences. The phylogenetic tree was constructed using molecular evolutionary genetic analysis program (MEGA, ver.7.0) with the minimum evolution algorithm. The evolutionary distances were computed using Kimura 2-Parameter method.
